# Mucoepidermoid carcinoma cured by a combination of high‐frequency snare and photodynamic therapy: A case report

**DOI:** 10.1111/1759-7714.14861

**Published:** 2023-03-16

**Authors:** Kentaro Tamura, Keigo Uchimura, Hideaki Furuse, Tatsuya Imabayashi, Yuji Matsumoto, Takaaki Tsuchida

**Affiliations:** ^1^ Department of Endoscopy, Respiratory Endoscopy Division National Cancer Center Hospital Tokyo Japan; ^2^ Department of Thoracic Oncology National Cancer Center Hospital Tokyo Japan; ^3^ Division of Respiratory Diseases, Department of Internal Medicine The Jikei University School of Medicine Tokyo Japan

**Keywords:** bronchial tumor, bronchoscopy, high‐frequency snare, mucoepidermoid carcinoma, photodynamic therapy

## Abstract

Mucoepidermoid carcinoma (MEC) is a rare salivary gland tumor, accounting for 0.2% of all lung tumors. The standard treatment for MEC of the primary bronchus is surgery, although intraluminal bronchoscopic treatment has recently become an option. A 68‐year‐old man presented with an asymptomatic bronchial tumor in the right intermediate bronchus. The tumor was resected using a high‐frequency snare (HFS) during bronchoscopy, and the specimen was pathologically diagnosed as low‐grade MEC. A residual lesion was detected in the resected area by autofluorescence imaging. The tumor appeared to be localized within the subepithelial layer without metastases, and photodynamic therapy (PDT) was performed as a local treatment. The patient had no recurrence for 18 months. PDT is effective and safe for patients with centrally located early‐stage lung cancer, but there are few reports of its use for rare tumors, such as MEC. In this case, PDT allowed for local control and avoided surgery, including bronchoplasty, for MEC. Combined treatment of tumor reduction by HFS and PDT of the residual lesion may be an optimal treatment for MEC of the bronchus.

## Graphical abstract

We present a case of low‐grade mucoepidermoid carcinoma (MEC) of the primary bronchus that was successfully cured with photodynamic therapy (PDT) for a residual lesion after resection of a bronchial tumor with high‐frequency snare (HFS) under flexible bronchoscopy. The combination of HFS and PDT may be an optimal treatment for MEC of the bronchus to avoid surgery.

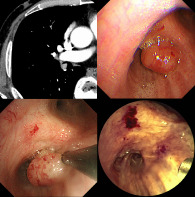

## INTRODUCTION

Pulmonary mucoepidermoid carcinoma (MEC) is classified as a salivary gland tumor based on the fifth edition of the World Health Organization classification, composed of mucin‐secreting cells, squamoid cells, and intermediate‐type cells.^1^ Based on morphology, MEC is classified as low or high grade, with most being low grade.^2^ MEC is rare, accounting for 0.2% of all lung tumors, and often occurs in central airways,^3^, ^4^ therefore surgery with bronchoplasty is often required for complete resection of MEC.^2^, ^4^, ^5^, ^6^, ^7^, ^8^ Recently, the utility and safety of intraluminal bronchoscopic treatments (IBTs), such as high‐frequency snare (HFS), argon plasma coagulation (APC), neodymium‐doped yttrium‐aluminum‐garnet (Nd:YAG) laser, and photodynamic therapy (PDT), have been reported for MEC as well as for central‐type early‐stage lung cancer (CELC).^9^, ^10^, ^11^, ^12^, ^13^

We report a case of MEC of the bronchus cured by the combination of tumor reduction using HFS and PDT to the residual tumor.

## CASE REPORT

**FIGURE 1:**
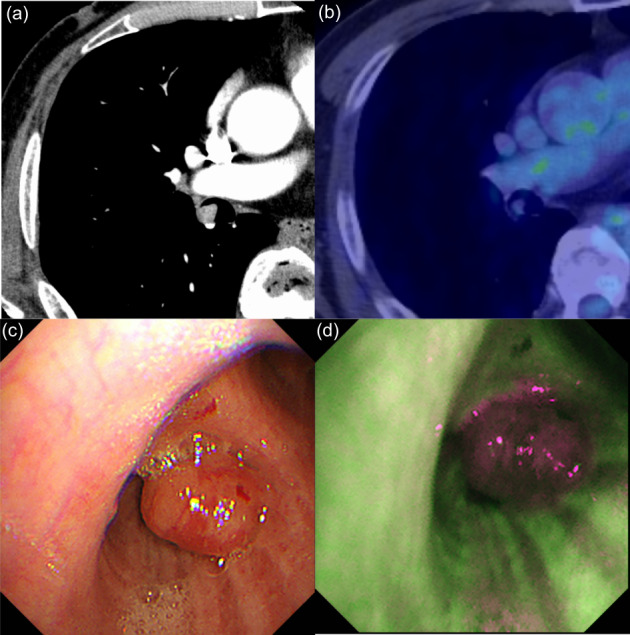
Chest computed tomography (CT), ^18^F‐fluorodeoxyglucose positron emission tomography‐CT (FDG‐PET‐CT), and bronchoscopic findings before treatments. Chest CT showed a bronchial tumor with a maximum diameter of 13 mm in the right intermediate bronchus (a). FDG‐PET‐CT showed that the maximum standardized uptake value of the tumor was 1.63 (b). White‐light bronchoscopy showed a polypoid tumor in the right intermediate bronchus (c). The tumor appeared magenta on autofluorescence bronchoscopy (d).

**FIGURE 2:**
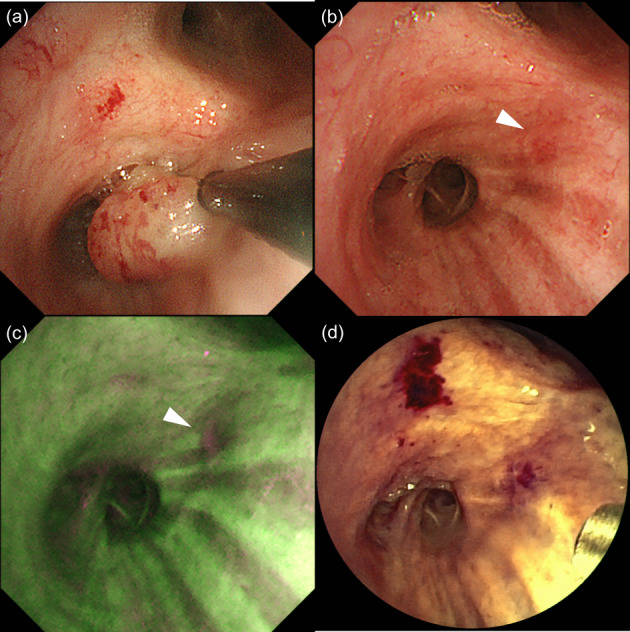
Bronchoscopic findings during intraluminal bronchoscopic treatments. The bronchial tumor was resected using a high‐frequency snare without electrification (a). After bronchoscopic resection, white‐light bronchoscopy did not show any elevated lesion at the root of the tumor (arrowhead) (b), but autofluorescence bronchoscopy showed magenta coloring (arrowhead), suggestive of residual tumor in the resected area (c). Photodynamic therapy was performed on the residual tumor (d).

**FIGURE 3:**
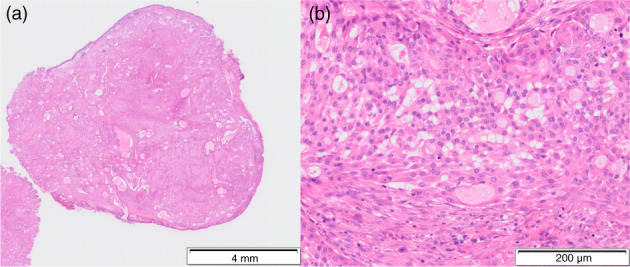
Histopathological findings of resected bronchial tumor. The resected tumor surrounded by the existing bronchial epithelium (a) was composed of squamoid cells, mucin‐secreting cells, and intermediate‐type cells (b), resulting in a diagnosis of low‐grade mucoepidermoid carcinoma.

A 69‐year‐old man without any symptoms was referred to us because of a bronchial tumor with a maximum diameter of 13 mm in the right intermediate bronchus detected on chest computed tomography (CT) (Figure 1a). The patient had a smoking history of 38 pack‐years, pulmonary emphysema, chronic kidney disease, and poorly controlled type 2 diabetes mellitus. ^18^F‐fluorodeoxyglucose positron emission tomography‐CT showed that the maximum standardized uptake value of the tumor was 1.63 (Figure 1b). Radiological findings showed no metastases or invasion beyond the bronchial walls. White‐light bronchoscopy (1TQ290; Olympus) showed a polypoid lesion in the right intermediate bronchus (Figure 1c), which appeared as magenta on autofluorescence bronchoscopy (AFB) (F260; Olympus) (Figure 1d). The tumor, which was resected using HFS (SD‐5L‐1; Olympus) without electrification (Figure 2a), was composed of squamoid cells, mucin‐secreting cells, and intermediate‐type cells with *MAML2* gene rearrangement on fluorescence in situ hybridization and was diagnosed as low‐grade MEC (Figure 3). The TNM classification was cT1bN0M0 and cStage IA2.

**FIGURE 4:**
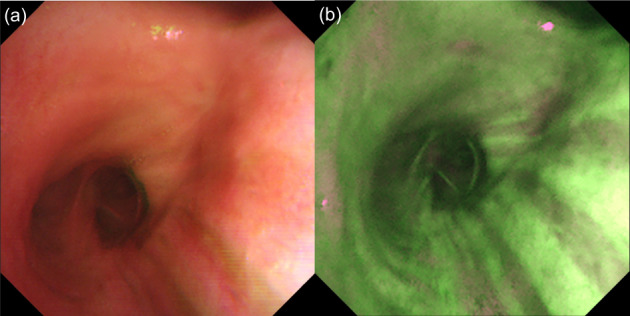
Bronchoscopic findings after intraluminal bronchoscopic treatments. White‐light bronchoscopy (a) and autofluorescence bronchosocpy (b) showed no recurrent findings.

Two weeks after bronchoscopic resection, AFB showed the root of the tumor to be magenta, suggesting a residual tumor (Figure 2b,c). The extent of invasion of the residual lesion was not confirmed using radial probe‐endobronchial ultrasonography. PDT was performed as a local treatment. Four hours after intravenous administration of mono‐L‐aspartyl chlorine e6 (NPe6) (40 mg/m^2^), a residual lesion was irradiated with a 664‐nm laser and a directional quartz fiber (straight‐type power density 150 mW/cm^2^, energy level 100 J/cm^2^) for 11 min and 7 s (Figure 2d). No adverse events occurred after PDT. No recurrence was observed 2, 6, 12, and 18 months after PDT on bronchoscopy (Figure 4a,b). Forceps biopsy of the irradiated area was performed at 12 months after PDT and the histological findings showed no evidence of malignancy.

## DISCUSSION

We present a case of MEC of the primary bronchus that was cured using a combination of HFS and PDT under flexible bronchoscopy without surgery.

Treatment for MEC of the bronchus is mainly lobectomy or sleeve resection, with a 5‐year overall survival rate of 86–87%.^4^, ^5^ Younger age, female sex, early stage, small size, low histological grade, presence of *MAML2* fusion gene, and complete resection are associated with a good prognosis.^2^, ^4^, ^5^, ^6^, ^7^, ^8^, ^14^ Surgery is superior to IBTs as it can provide whole‐tumor and lymph node specimens for diagnosis, and recent guidelines recommend IBTs only for patients with intolerance of surgery.^15^ However, complications associated with bronchoplasty cannot be ignored compared to those with IBTs, and even wedge resection of the bronchus, which is considered less invasive, has morbidity and mortality rates of 11.8% and 5.9%, respectively.^16^

PDT is an effective and safe IBT that can preserve pulmonary function, and the complete response rate for patients with CELC < 20 mm in diameter has been reported as 84.6–94.0%.^17^, ^18^ Most adverse events caused by PDT are cutaneous toxicities, including photosensitivity, which are nonsevere and manageable,^17^ therefore patients with MEC may prefer PDT over surgery because of its minimal invasiveness.

Several cases of bronchial MEC cured using APC or Nd:YAG laser have been reported,^10^, ^11^, ^12^, ^13^ as well as by PDT after failed treatment with Nd:YAG laser.^9^ It is currently unclear whether PDT or other IBTs are more suitable for the treatment of MEC. PDT with NPe6 may be effective for centrally located lung cancer with extracartilaginous invasion when combined with tumor reduction by other IBTs.^18^ PDT can be performed repeatedly, and PDT in combination with other IBTs may be a treatment option for MEC of the bronchus because of its efficacy and safety. However, as the recurrence pattern of MEC is often local,^4^ systemic CT and bronchoscopy should be performed regularly after IBTs.

The importance of accurately assessing the peripheral margins of bronchial tumors for successful PDT has been reported.^18^ HFS with electrification has a hemostatic effect during tumor resection, but also incinerates the airway epithelium around the tumor, obscuring the peritumoral margin. Tumor reduction using HFS without electrification, as in this case, may lead to more accurate PDT. Therefore, bronchoscopists should carefully choose whether or not to energize the HFS when performing PDT after tumor resection.

In conclusion, we report a case of MEC of the primary bronchus that was treated with a combination of HFS and PDT, without surgery. Even rare tumors such as MEC can be cured by IBTs alone with appropriate case selection.

## AUTHOR CONTRIBUTIONS

Kentaro Tamura drafted this manuscript. Keigo Uchimura, Hideaki Furuse, Tatsuya Imabayashi, Yuji Matsumoto, and Takaaki Tsuchida were responsible for this case. Keigo Uchimura, Hideaki Furuse, Tatsuya Imabayashi, Yuji Matsumoto, and Takaaki Tsuchida helped in drafting the manuscript. All authors have read and approved the final manuscript.

## FUNDING INFORMATION

This work was supported by JSPS KAKENHI (grant number JP22K15698).

## CONFLICT OF INTEREST STATEMENT

The authors declare that they have no competing interests.
